# Synaptic vesicles are “primed” for fast clathrin-mediated endocytosis at the ribbon synapse

**DOI:** 10.3389/fnmol.2014.00091

**Published:** 2014-12-01

**Authors:** Ilaria Pelassa, CongJian Zhao, Mathias Pasche, Benjamin Odermatt, Leon Lagnado

**Affiliations:** Medical Research Council Laboratory of Molecular Biology, Neurobiology DivisionCambridge, UK

**Keywords:** synapse, vesicle, endocytosis, clathrin, zebrafish

## Abstract

Retrieval of synaptic vesicles can occur 1–10 s after fusion, but the role of clathrin during this process has been unclear because the classical mode of clathrin-mediated endocytosis (CME) is an order of magnitude slower, as during retrieval of surface receptors. Classical CME is thought to be rate-limited by the recruitment of clathrin, which raises the question: how is clathrin recruited during synaptic vesicle recycling? To investigate this question we applied total internal reflection fluorescence microscopy (TIRFM) to the synaptic terminal of retinal bipolar cells expressing fluorescent constructs of clathrin light-chain A. Upon calcium influx we observed a fast accumulation of clathrin within 100 ms at the periphery of the active zone. The subsequent loss of clathrin from these regions reflected endocytosis because the application of a potent clathrin inhibitor Pitstop2 dramatically slowed down this phase by ~3 fold. These results indicate that clathrin-dependent retrieval of synaptic vesicles is unusually fast, most probably because of a “priming” step involving a state of association of clathrin with the docked vesicle and with the endosomes and cisternae surrounding the ribbons. Fluorescence correlation spectroscopy (FCS) and fluorescence recovery after photobleaching (FRAP) showed that the majority of clathrin is moving with the same kinetics as synaptic vesicle proteins. Together, these results indicate that the fast endocytic mechanism operating to retrieve synaptic vesicles differs substantially from the classical mode of CME operating via formation of a coated pit.

## Introduction

The classical mode of clathrin-mediated endocytosis (CME) is the major pathway by which cells internalize components of the surface membrane (Conner and Schmid, 2003; Jung and Haucke, 2007; Heerssen et al., 2008). Imaging methods have allowed CME to be investigated in real-time, particularly in the context of receptor/agonist uptake from the cell surface, which occurs on the time-scale of ~20–40 s (Loerke et al., 2009). These relatively slow kinetics have caused some to question whether CME is also an important mechanism of synaptic vesicle retrieval (Pyle et al., 2000; Harata et al., 2001; Sara et al., 2002). Nonetheless, a number of studies have demonstrated that clathrin-dependent retrieval of synaptic vesicles can occur on the time-scale of 10 s, both in ribbon-type synapses of sensory neurons and in conventional synapses of central mammalian neurons (Neves and Lagnado, 1999; Jockusch et al., 2005; Granseth et al., 2006; Balaji and Ryan, 2007; Saheki and De Camilli, 2012). More recent findings identified an “ultrafast” mechanism of retrieval at mouse hippocampal synapses and at *Caenorhabditis elegans* neuromuscular junctions (Watanabe et al., 2013a). During ultrafast endocytosis synaptic vesicles are retrieved within 50–100 ms after vesicle fusion and endocytic pits lack the stereotypical clathrin coat. Knock-down experiments demonstrated that ultrafast endocytosis acts in a clathrin-independent manner (Watanabe et al., 2014). Moreover it was also shown that clathrin is involved in regenerating synaptic vesicles directly from endosomes (Watanabe et al., 2014). These observations support the idea that a “souped-up” mode of CME acts at the synaptic terminal (Conner and Schmid, 2003). We now need to understand the mechanistic differences that make clathrin-dependent retrieval of synaptic vesicles an order of magnitude faster than classical CME.

A direct method for investigating the molecular dynamics of endocytosis in living cells is total internal reflection fluorescence microscopy (TIRFM), which allows the movement of fluorophores within ~50 nm of the surface membrane to be imaged in real-time (Merrifield et al., 2002, 2005). Several steps in the formation of a clathrin-coated vesicle have been visualized by TIRFM, and it appears that the process is rate-limited by the assembly of the clathrin coat around the invagination (or “pit”) at the surface membrane, which occurs over ~1–2 min (Loerke et al., 2009). Subsequently, departure of the clathrin-coated vesicle occurs on the time-scale of ~10 s. In contrast, little is known of the dynamics of clathrin association and dissociation at the surface of the synaptic terminal during calcium-triggered fusion of synaptic vesicles (Mueller et al., 2004). Is the recruitment of clathrin to a synaptic vesicle faster than recruitment to a coated pit?

Here we present the first use of TIRFM to investigate clathrin dynamics associated with synaptic activity. To achieve this, we created transgenic zebrafish expressing fluorescent fusion proteins at ribbon synapses of sensory neurons (Odermatt et al., 2012; Nikolaev et al., 2013). Using retinal bipolar cells isolated from these fish, we imaged clathrin light chain A-EGFP in temporal relation to exocytosis and endocytosis assayed using sypHy (Granseth et al., 2006). We observed that at the ribbon synapse clathrin is recruited within 100 ms from the beginning of the stimulus. In addition, we provided a number of pieces of evidence such as fluorescence recovery after photobleaching (FRAP) and fluorescence correlation spectroscopy (FCS) indicating that the majority of clathrin in the terminal at resting conditions is not moving as a “free” molecule but it follows closely the kinetics of synaptic vesicle proteins. These results indicate that the retrieval of synaptic vesicles is faster than classical CME because it is not rate-limited by the recruitment of clathrin. Instead, is possible that vesicles in a resting synapse are “primed” for fast clathrin-dependent retrieval before fusion is triggered.

## Materials and methods

### Animals

Zebrafish (*Danio rerio*) were maintained according to Home Office regulations. Fish were maintained as described by Nusslein-Volhard and Dahm (2002) using a 14:10 h light dark cycle at 28°C. Transgenic animals were generated in a mixed genetic background from fish originally purchased from a local aquatic supplier (Scotsdales line), using plasmids taking advantage of the *I-SceI* meganuclease co-injection protocol (Thermes et al., 2002). Most experiments were carried out on fish heterozygous for the inserted reporter genes to avoid possible unwanted side effects of homozygous genomic insertions. The following lines were generated for this study, with the official nomenclature in brackets: Rib:sypHy (*Tg(-1.8ctbp2:SYPHY)lmb2)*; Rib:Synaptophysin-EGFP (*Tg(-1.8ctbp2:sypb-EGFP)lmb4)*; Rib: Clathrin-EGFP *(Tg(-1.8ctbp2:clta-EGFP)lmb5)*; Rib:Dyn2-EGFP (*Tg(-1.8ctbp2:dnm2-EGFP)lmb8*); Rib:Clathrin-mCherry (*Tg(-1.8ctbp2:clta-mCHERRY)lmb6)*; Rib:Rib-mCherry (C1-3) (*Tg(-1.8ctbp2:mCHERRY-ctbp2)lmb7)* (Thermes et al., 2002).

### Transgenic zebrafish lines

For the generation of injection plasmids containing the zebrafish *ribeye a (ctbp2)* promoter for the retina bipolar cell work, we used the I-SceI *ribeye a* promoter plasmid (Odermatt et al., 2012) based on the pBluescript® II KS+ phagemid (Stratagene).

To generate the *ribeye a:clathrin-EGFP/mCherry* injection plasmids, in a first step the cDNA coding sequence of zebrafish clathrin light chain a (*clta*; ZFIN ID: ZDB-GENE-040426-1986; IMAGE clone: 4144130) was PCR cloned into the *XhoI* and *BamHI* sites of the pEGFP-N1 vector (BD Biosciences, Clontech) using the primers Zf clathrin forward and reverse (see Supplementary Table 1). In a second step, from this vector, the clathrin-EGFP sequence followed by a SV40 polyA domain from *NheI* to *SspI* was cloned into the *SpeI* and *EcoRV* sites of the I-SceI *ribeye a* promoter plasmid. Finally for the I-SceI *ribeye a:clathrin-mCherry* injection plasmid EGFP was replaced by the coding region of mCherry from the pmCherry-N1 vector (BD Biosciences, Clontech) using *BamHI* and *NotI* sites.

For the *ribeye a:synaptophysin-EGFP* injection plasmid, accordingly the coding sequence of zebrafish synaptophysin b (*sypb*; ZFIN ID: ZDB-GENE-040718-205; IMAGE clone: 7287035) was PCR cloned into the *XhoI* and *BamHI* sites of the pEGFP-N1 vector (BD Biosciences, Clontech) using the primers Zf synaptophysin forward and reverse (see **Supplementary Table 1**). In a second step again, from this vector, the synaptophysin-EGFP sequence followed by a SV40 polyA domain from *NheI* to *SspI* was cloned into the *SpeI* and *EcoRV* sites of the I-SceI *ribeye a* promoter plasmid.

The *ribeye a:mCherry-ribeye a* injection plasmid was directly generated from adult wild type zebrafish retina mRNA after reverse transcription into cDNA with oligo(dT) primers. The coding sequence of zebrafish ribeye a, also called C-terminal binding protein 2 (ctbp2; ZFIN ID: ZDB-GENE-010130-2; transcript variant 1) was PCR cloned into the *XhoI* and *EcoRI* sites of the pmCherry-C1 vector (BD Biosciences, Clontech) using the primers Zf ribeye a forward and reverse (see **Supplementary Table 1**). In this case, from this vector, the mCherry-ribeye a sequence followed by a SV40 polyA domain from *NheI* to *AvrII* was cloned into the SpeI site of the I-SceI *ribeye a* promoter plasmid and checked for proper orientation.

The generation of the *ribeye a:sypHy* injection plasmid and a stable transgenic line of fish (*Tg(-1.8ctbp2:sypHy)lmb)* expressing sypHy under the zebrafish ribeye a promoter has been described previously (Odermatt et al., 2012).

The *ribeye a:dynamin2-EGFP* injection plasmid was also generated from retina mRNA after reverse transcription into cDNA with oligo(dT) primers. The coding sequence of zebrafish dynamin2 (dnm2; ZFIN ID: ZDB-GENE-050913-84) was PCR cloned into the *AfeI* and *AgeI* sites of the *ribeye a:clathrin-EGFP* vector (described above) using the primers Zf dynamin2 forward and reverse (see **Supplementary Table 1**). Hereby zebrafish *clathrin light chain a* was replaced by the coding region of zebrafish dynamin2 (*PmlI* to *AgeI*) thereby fused to EGFP. By subsequent sequencing we found that 3 out of 4 *ribeye a:dynamin2-EGFP* injection plasmids we had cloned this way showed an additional 12 base pairs stretch *ggt-gaa-atc-ctg* (bp 1543 - 1554; starting from *atg*) compared to the cDNA GenBank sequence BC097134.1. These base pairs are coding for the additional 4 amino acids GEIL (Aa 515 -518) which we assumed to be a retina specific transcript variant and therefore used for the further generation of the transgenic fish line (*Tg(-1.8ctbp2:dnm2-EGFP)lmb8)*.

### Bipolar cell isolation

Bipolar cells were dissociated from the retinae of adult transgenic zebrafish by papain digestion and mechanical trituration as described previously (Burrone and Lagnado, 1997). Retinae were incubated in papain (30 U/ml Papain from Carica Papaya, 76218, Fluka) for 45–60 min at room temperature in Leibovitz's medium adjusted to 260 mOsm with H_2_O (21083, GIBCO, Invitrogen) containing 5.5 mM cysteine (Sigma). pH was adjusted to 7.0 with NaOH. The digestion times and papain concentrations varied depending on the batch of the enzyme. After digestion, retinae were mechanically triturated with fire-polished glass pipettes in 0.5 mM bovine serum albumine (BSA) in Leibovitz's Medium and plated on 16 mm high refractive index coverslips (Plan Optik AG, Elsoff, Germany) coated with 3 μM concanavalin A (C-2631, Sigma) and 2 mg/ml poly-L-lysine (P1399, Sigma).

### Total internal reflection fluorescence microscopy (TIRFM)

TIRFM was performed with an inverted microscope (Zeiss Axiovert S100TV) modified for TIRFM using a 100× oil objective 1.65 NA (Olympus). EGFP excitation was provided by an Argon Ion laser at 488 nm wavelength (50 mW Laserphysics, UK) and mCherry excitation with a solid state laser at 561 nm wavelength (50 mW Cobolt Jive). The laser intensity was modified with Neutral Density (ND) filters in an automatic optical filter changer Lambda 10-2 (Sutter). The two laser lines were combined in the same path with a dichroic mirror (DMLP505, Thorlabs) and focused with a visible achromatic lens (AC127-025-A, Thorlabs). The evanescent field was generated by positioning of the laser beam to a total reflection angle through a motorized protected silver mirror. To perform simultaneous double color imaging an ET GFP-mCherry dual beamsplitter filter set was chosen (59022 Chroma Technology Corp. Bellows Falls, USA). Images were recorded with an EM CCD camera (Hamamatsu).

Cells were stimulated using a modified pressure clamp (HSPC-1, ALA Scientific Instruments, Farmingdale, NY, USA) that allowed fluid jets to be applied with precise timing. A depolarizing solution was applied through a glass pipette with a diameter of ~1 μm. Stimulus durations were either 0.5 or 3 s, and the pipette was placed ~2 μm from the terminal. The depolarizing solution was sufficient to depolarize the membrane to ~0 mV and contained (mM): 70 NaCl, 50 KCl_2_, 2.5 CaCl_2_, 1 MgCl_2_, 10 Glucose, and 10 Hepes hemisodium.

For experiments with the endocytosis inhibitor Pitstop2 (ab120687, abcam, Cambridge, UK), solutions were modified to contain DMSO in control medium and 30 μM Pitstop2 dissolved in DMSO. Cells were stimulated twice before incubation with Pitstop2 for 5 min.

### TIRFM-FRAP

We applied FRAP in combination with TIRFM, to bleach selectively only the molecules near the plasma membrane. An automatic filter wheel, controlled by an iVision software script, altered the illumination of the sample. The change in mean fluorescence of the footprint was calculated in ImageJ. Data analysis was carried out in Igor Pro 6.2 and the recovery curve was fitted with a single exponential function and τ was calculated. Data were normalized to zero at the fluorescence value after bleaching and to the maximum value of fluorescence and then averaged thereby calculating standard errors.

### TIRF image analysis

Processed movies were analyzed in Igor Pro using a collection of routines called Semi-Automated Routines for Functional Image Analysis (SARFIA) developed in our laboratory (Dorostkar et al., 2010). Regions of interest (ROI) were defined with segmentation by thresholding and to improve detection of units at low contrast the image was transformed using the Laplace Operator. The ROIs were numbered and stored in a matrix and a flowchart with time series of all ROIs was generated automatically. Responding ROIs were selected with an unbiased approach: traces were selected by a threshold-crossing algorithm based on the calculation of average standard deviation on the baseline. To calculate the distance of vesicle fusion and retrieval from the center of the ribbons we wrote a procedure in Igor Pro that calculates the distance of the center of mass of the fusion or retrieval from the center of mass of the nearest ribbon.

The reliability in defining hot-spots of sypHy or clathrin LCa-mCherry varied between terminals, and more diffuse signals were often observed. The latter is consistent with the observation that vesicles do not fuse exclusively at the active zone, but are also capable of fusing at lower rates at sites remote from ribbons (Zenisek, 2008; Zampighi et al., 2011). The kinetics of the signal from each reporter were therefore quantified simply by measuring the relative change in fluorescence over a single ROI encompassing the footprint at rest.

### Confocal microscopy and FCS

All FCS and FCCS measurements were carried out on a Zeiss LSM 780 confocal microscope (63×/1.4NA oil objective lens, 488 nm/25 mW max. and 561 nm/15 mW max. laser lines). The GaAsP spectral detector was set to 490–550 nm for EGFP and 595–651 nm for mCherry and mRFP. The correlator binning time was 0.2 μs. Laser intensity was set for each experiment due to varying fluorophore concentrations and brightness to avoid bleaching during experiments. The measurement times of FCS was 10 s for molecules diffusing rapidly (R110, purified GFP, mCherry-RFP, mRFP in HEK cells; repetition was 5–10 times) and 50 or 200 s for proteins displaying slower motion (GluR2, clathrin, synaptophysin, AP180). A 3 s prebleaching step with 10 times experimental laser intensity was applied routinely.

### Image analysis

Images and FCS data were analyzed using Carl Zeiss ZEN software and custom-written macros in Igor Pro software.

(1) $$G_{ac}\left(\tau \right)=\frac{\langle \delta F\left(t\right)\cdot \delta F\left(t+\tau \right)\rangle }{{\langle F\left(t\right)\rangle }^{2}},\text{ where }\delta F\left(t\right)=F\left(t\right)-\langle F\rangle$$

FCS data from fast diffusion species (including Rohdamin110, EGFP, mCherry, cyto EGFP, cytomRFP, dynamine2 EGFP, endophiline- EGFP) were fitted with an autocorrelation function that describes a three dimensional diffusion model, above species except R110 were analyzed with a blinking component as well:

(2.1) $$\begin{array}{l} G_{ac}\left(\tau \right)=\left(1+\frac{T}{1-T}*e^{-\tau /\tau_{T}}\right)*\frac{\gamma }{N}\cdot \frac{1}{\left(1+\left(\frac{\tau }{\tau_{D}}\right)\right)}\\ \;\cdot \;\frac{1}{\sqrt{\left(1+\left({\left(\frac{r_{0}}{z}\right)}^{2}{\left(\frac{\tau }{\tau_{D}}\right)}^{a}\right)\right)}}+1 \end{array}$$

(2.2) $$G_{ac}\left(\tau \right)=\frac{\gamma }{N}\cdot \frac{1}{\left(1+\left(\frac{\tau }{\tau_{D}}\right)\right)}\cdot \frac{1}{\sqrt{\left(1+\left({\left(\frac{r_{0}}{z}\right)}^{2}{\left(\frac{\tau }{\tau_{D}}\right)}^{a}\right)\right)}}+1\;\;\;\;\;\;\;\;\;$$

In some set clathrin FCS data two-component fitting was applied:

(3) $$\begin{array}{l} G_{ac}\left(\tau \right)=\left(1+\frac{T}{1-T}*e^{-\tau /\tau_{T}}\right)*\frac{\gamma }{N}\cdot (Y*\frac{1}{\left(1+\left(\frac{\tau }{\tau_{D1}}\right)\right)}\\ \;+\;\left(1-Y\right)*\frac{1}{\left(1+\left(\frac{\tau }{\tau_{D2}}\right)\right)}) \end{array}$$

FCCS:

(4) $$G_{cc}\left(\tau \right)=\frac{\langle \delta P1\left(t\right)\cdot \delta P2\left(t+\tau \right)\rangle }{\langle P1\left(t\right)P2\left(t\right)\rangle }$$

Diffusion coefficient:

(5.1) $$D=\frac{r_{0}{}^{2}}{4\tau_{D}}$$

(5.2) $$D=\frac{r_{0}{}^{2}}{2\tau_{D}}$$

***N_bound_*** species was calculated using below equation (Slaughter et al., 2007)

(6) $$\begin{array}{l} N_{\mathrm{bound}}=\frac{N_{g}\left(N_{r}+Q\cdot N_{g}\right)}{N_{cc}}-Q\cdot N_{g}\\ \text{ Where }Q=\frac{{\mathrm{CPSM}}_{\mathrm{redchannel}\;488\mathrm{ex}}}{{\mathrm{CPSM}}_{\mathrm{redchannel}\;561\mathrm{ex}}} \end{array}$$

Where ***D*** is the diffusion coefficient of the fluorescent molecules, *t* is the time variable, and *r*_0_ is the radius of the detection volume in the experimental configuration. γ/*N* (*G*_0_) is the amplitude of the autocorrelation function at the y-intercept and is the inverse of the number of molecules (*N*) in the detection volume. (http://research.stowers-institute.org/microscopy/external/Technology/PCH/NBccCalculator.html)

### Western blot

Western Blot experiments were performed on total retina lysates from mouse, goldfish, and zebrafish using a common protein extraction (RIPA buffer). Clathrin protein was detected using clathrin light chain mouse monoclonal antibody diluted to 1:1000 from Synaptic Systems (Cat.n. 113011 Göttingen). Primary antibody was incubated overnight at 4°C. As control blot we used an anti-tubulin antibody (SIGMA). After incubation with HRP secondary antibody, the signal was detected with ECL reaction (Pierce, Thermo Scientific).

### Electrophysiology

Membrane capacitance measurements were performed as previously described. For details see Jockusch et al. (2005).

## Results

### Clathrin is compartimentalized with synaptic vesicles in a ribbon synapse

**Figure 1:**
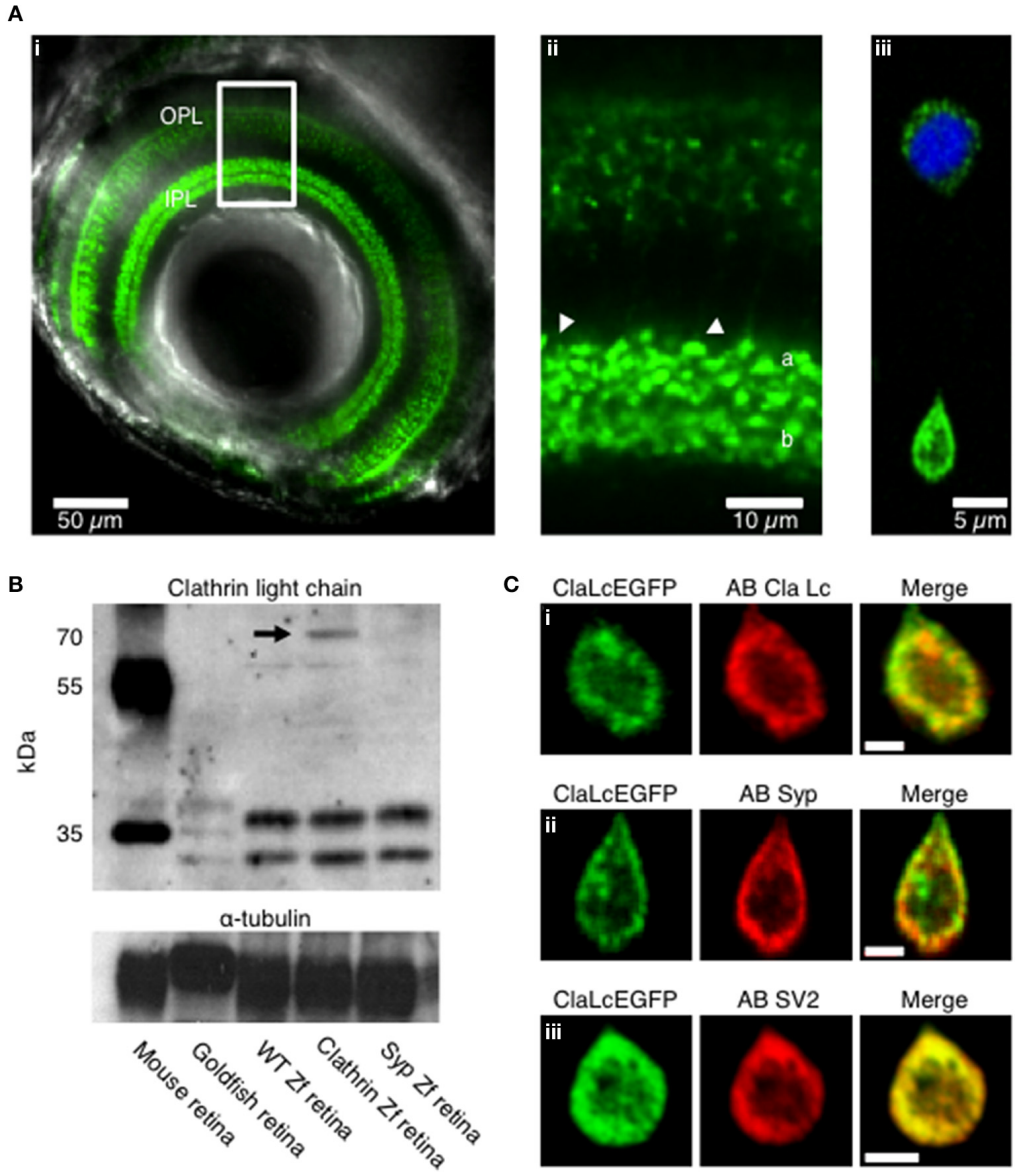
**Expression pattern of clathrin in transgenic zebrafish. (A)** Confocal image of the eye of a Ribeye:clathrin-EGFP larva (**i**, 5 dpf). Clathrin LCa-EGFP is expressed densely in the synaptic terminals of bipolar cells in the inner plexiform layer (**ii**, IPL). The right-hand panel is an example of a bipolar cell dissociated from such a fish **(iii)**. **(B)** Western Blot with a primary antibody directed against clathrin neuronal light chain. Protein bands at appropriate molecular weights were labeled in membrane homogenates of retinae from mouse, goldfish, wild-type zebrafish, Ribeye::ClathrinLCa-EGFP (RCE) zebrafish and Ribeye::synaptophysin-EGFP (RSE) zebrafish. The antibody against clathrin LCa recognizes a doublet at about 36–38 KDa in the mouse retina and a triple band in goldfish retina (Sherry and Heidelberger, 2005). In wild-type zebrafish and RSE retinae, the antibody recognizes two species at about 34–36 KDa. In RCE retinae a third band is detected is detectable at about 70 KDa, corresponding to the clathrin LCa-EGFP fusion protein (indicated by the black arrow). **(C)** Immunostaining of dissociated bipolar cells. **(i)** Distribution of Clathrin LCa-EGFP is similar to the endogenous protein (red). Clathrin LCa-EGFP co-localized with synaptophysin 1 **(ii)** and with SV2 **(iii)** Scale bars: 2 μm.

To investigate the role of clathrin in the retrieval of synaptic vesicles we generated transgenic zebrafish expressing the neuronal form of Clathrin Light Chain “a” (LCa) tagged with EGFP, under control of the *Ribeye a* promoter (Odermatt et al., 2012). First we examined by confocal microscopy the expression *in vivo* of clathrin in zebrafish larvae (5 days post fertilization). Clathrin was highly enriched in the synaptic terminals of bipolar cells projecting throughout the inner plexiform layer of the retina (IPL; Figures 1Ai,ii). Then we verified the expression at the level of dissociated bipolar cells, here clathrin is localized mainly at the synaptic terminal, low level of protein is present at the cell body around the nucleus (Figure 1Aiii, the nucleus is shown in blue Hoechst staining). Western blot analysis from total retina lysates show clathrin bands at appropriate molecular weights from mouse, goldfish, wild-type zebrafish, Ribeye::ClathrinLCa-EGFP (RCE) zebrafish and Ribeye::synaptophysin-EGFP (RSE) zebrafish. In RCE retinae a third band is detectable at about 70 KDa, corresponding to the clathrin LCa-EGFP fusion protein (indicated by the black arrow). This showed that the synaptic enrichment of LCa-EGFP was not an artifact of overexpression but reflected the normal distribution of clathrin within these neurons (Figure 1B).

Confocal sections of dissociated bipolar cells revealed that the large majority of clathrin was distributed toward the periphery of the terminal, in a zone extending about 2 μm from the plasma membrane (Figure 1Ci). This distribution mirrors the location of synaptic vesicles, as assessed by staining with FM dyes (Lagnado et al., 1996) or by electron microscopy (Holt et al., 2004; Matthews and Sterling, 2008). Further evidence for the compartimentalization of clathrin with synaptic vesicles was provided by immunostaining against the synaptic vesicle markers synaptophysin (Figure 1Cii; average Pearson correlation coefficient 0.63 ± 0.17, of *n* = 5 images, *p* < 0.004) and SV2 (Figure 1Ciii; correlation coefficient 0.97 ± 0.01, of *n* = 5 images, *p* < 0.004).

The central region of the terminal tends to exclude synaptic vesicles by volume, being densely occupied by mitochondria (Holt et al., 2004; Matthews and Sterling, 2008). To test whether the peripheral location of clathrin might reflect volume exclusion rather than a real association with synaptic vesicles, we also investigated the distribution of another endocytic protein involved in CME dynamin2. Dynamin2 is a GTPase which is involved in the scission events of endocytosed vesicles and generally cytosolic (Praefcke and McMahon, 2004). While Dynamin2 was distributed relatively uniformly, clathrin and synaptophysin, were concentrated toward the periphery (Supplementary Figures 1A,B). These static images therefore provide evidence that clathrin is compartimentalized in the same area as synaptic vesicles.

**Figure 2:**
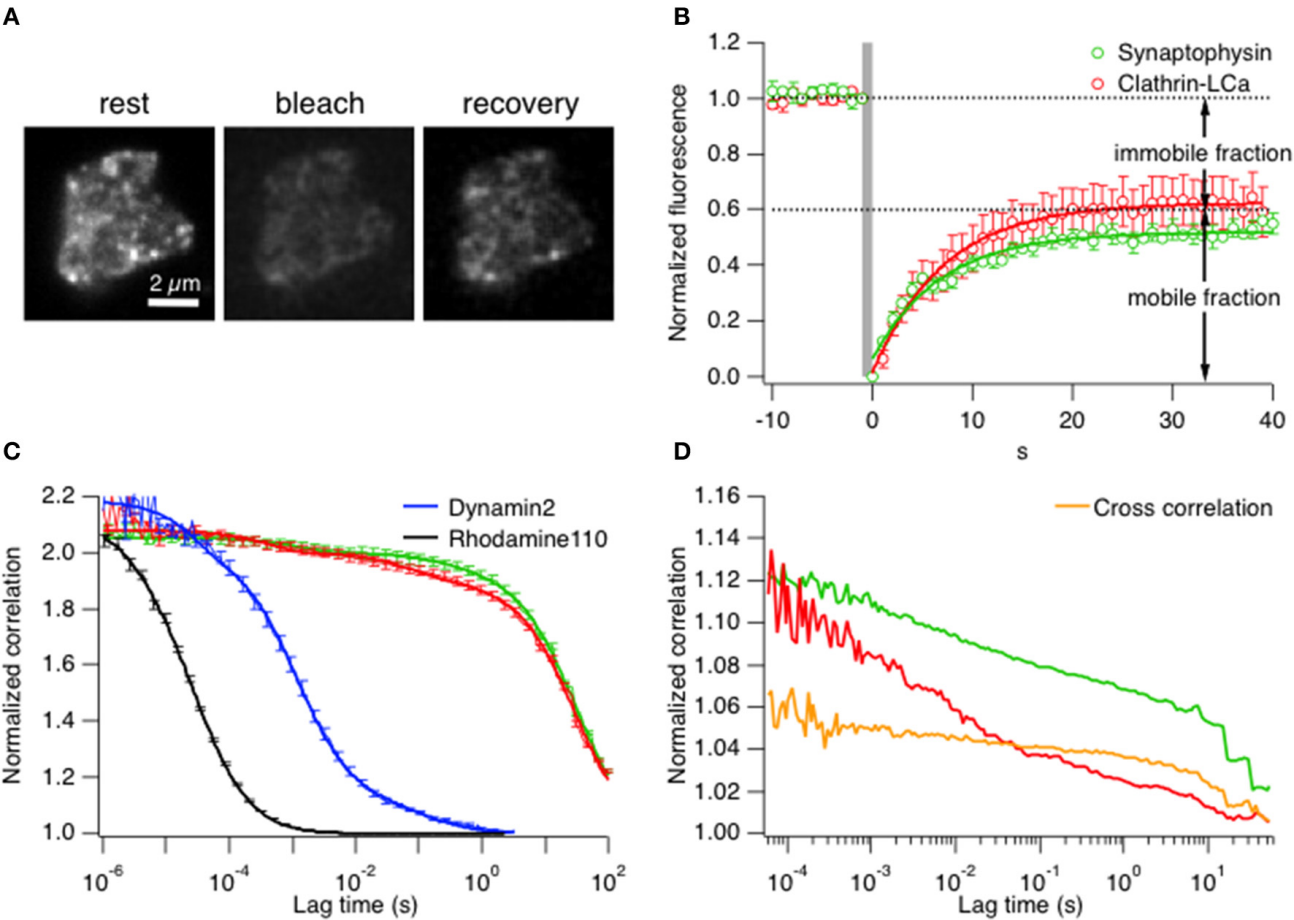
**Clathrin diffusion parallels synaptic vesicles in the ribbon synapse. (A)** TIRF–FRAP of a bipolar cell terminal expressing clathrin LCa-EGFP. **(B)** Time-course of fluorescence recovery after bleach of clathrin LCa-EGFP (red, *t* = 7.1 ± 0.3 s) and synaptophysin-EGFP (green, *t* = 8.1 ± 2.4 s), measured in separate experiments (s.e.m. error bars are displayed in the figure). **(C)** FCS measurements of correlation as a function of lag-time in bipolar cell terminals. The slower the diffusion coefficient, the higher the correlation at longer lag-times. Clathrin-LCa (red trace) and synaptophysin (green trace) have very similar diffusion coefficients (*D_Clathrin-LCa_* = 0.0096 μm^2^ s^−1^, *D_Syp-EGFP_* = 0.005 μm^2^ s^−1^). This function is compared with two smaller molecules, also measured in bipolar cell terminals: Rhodamine 110 (MW 336, black trace) moves with *D* = 400 μm^2^ s^−1^ while Dynamin2-EGFP (MW 123 kDa, blue trace) moves with *D* = 8 μm^2^ s^−1^. (s.e.m. error bars are displayed in the figure). **(D)** FCCS measurements to assess the degree of cross-correlation between synaptophysin-EGFP and clathrin LCa-mCherry. 66.6% of clathrin travels with similar kinetics of synaptic vesicles in bipolar cell terminals.

Subsequently measurements were made in live cells by comparing the mobility of clathrin LCa with synaptophysin, a transmembrane protein that is strongly enriched in synaptic vesicles and can therefore be used as a marker for this intracellular compartment. The first comparisons of mobility were by TIRF–FRAP (fluorescence recovery after photobleaching; see Materials and Methods). Figure 2A shows an example footprint from a bipolar cell terminal expressing clathrin LCa-EGFP at rest and then after bleaching of fluorophores in the evanescent field. Collected results from 7 cells exposed to a 1 s bleach are shown in Figure 2B. The mobile fraction of clathrin (~60%) recovered with an average time-constant of 7.1 ± 0.3 s. When the analogous measurement was made in cells expressing synaptophysin-EGFP, 50% of the fluorescence recovered with a time constant of 8.1 ± 2.4 s (*n* = 6), indicating that most of the clathrin in the terminal had a similar mobility to synaptic vesicle proteins.

A second approach for assessing clathrin mobility was fluorescence correlation spectroscopy (FCS; see Materials and Methods). FCS allows the direct *in vivo* measurement of diffusion kinetics within a native cellular environment by simply parking the laser beam at a desired position within the cell. Therefore, it is crucial to consider the exact location of the beam. Since we were interested in mobility of vesicles and clathrin at active zones, the beam was parked relatively close to the inner leaflet of the plasma membrane. Fluctuations of fluorescence intensity were monitored for a certain time window to obtain the characteristic residence time (τ_*d*_) at rest of a synaptic vesicle, assessed using synaptophysin-EGFP (Figure 2C, green trace). Figure 2C shows that the characteristic residence time (τ_*d*_) at rest of a synaptic vesicle, assessed using synaptophysin-EGFP (green trace), was 4.61 s yielding a diffusion coefficient of *D* = 0.005 μm^2^ s^−1^ (*n* = 7 cells). More than 95% of clathrin LCa-EGFP (Figure 2C, red trace) displayed similarly slow diffusion (τ_*d*_ = 2.3 s, *D* = 0.0096 μm^2^ s^−1^). The small remainder of clathrin LCa-EGFP was three orders of magnitude more mobile (*D* = 4 μm^2^ s^−1^), presumably reflecting the fraction that was “free”. The diffusion coefficient is expected to vary as the cube root of the MW, so these measurements are broadly consistent with the idea that all the clathrin light chain is present in the form of triskelia (MW = 650 kD). As a control for these measurements this function is compared with Rhodamine 110 (MW 336, black trace) and Dynamin2-EGFP (MW 123 kDa, blue trace), measured, respectively in PBS and in bipolar synaptic terminals. Rhodamine 110 moves with *D* = 400 μm^2^ s^−1^ while dynamin2 moves with *D* = 8 μm^2^ s^−1^, dramatically different from clathrin and synaptophysin. For further controls and calibration see Supplementary Figures 3A–D. Finally, we tested how far clathrin LCa-mCherry and synaptophysin-EGFP molecules moved together using fluorescence cross-correlation spectroscopy (FCCS) (Figure 2D). About 45% of synaptophysin particles moved together with clathrin molecules, and 67% of clathrin particles with synaptophysin (*n* = 9 terminals), which was cross-confirmed by dual FRAP of clathrin LCa-mCherry and synaptophysin-EGFP with a similar recovery constant (Supplementary Figure 3E).

Together, the results in Figures 1, 2 indicate that significantly less than half of clathrin triskelia in this ribbon synapse are free in the cytoplasm: the majority has slow diffusion comparable with synaptic vesicles, indicating that they might be bound or immobilized on active zone-surrounding structures through the volume of the presynaptic terminal.

### Depolarization triggered fast and transient accumulation of clathrin around the active zone

**Figure 3:**
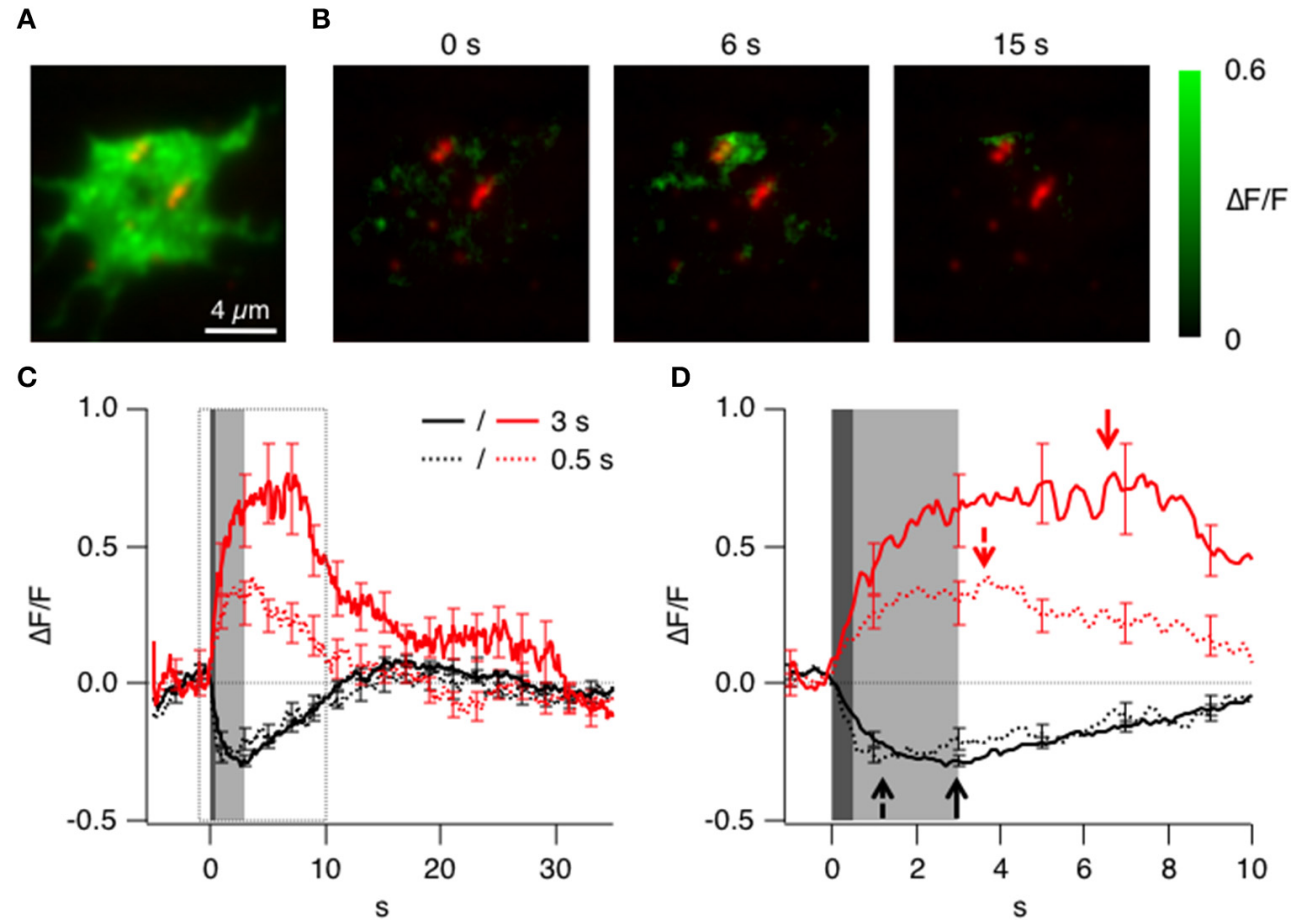
**Triggered accumulation of clathrin at the active zones. (A)** TIRF image of the synaptic terminal of a bipolar cell isolated from a fish generated by crossing Ribeye:LCa-EGFP and Ribeye:Ribeye-mCherry fish. **(B)** Images showing the relative change in fluorescence of LCa-EGFP at 0, 6, and 15 s delay relative to the start of a depolarizing stimulus lasting 3 s. Note the accumulation of LCa-EGFP around the two ribbons to the top of the footprint. **(C)** Average timecourse of clathrin accumulation (red) and loss (black) triggered by stimuli lasting 0.5 s (dotted) and 3 s (solid). **(D)** Responses in C shown on an expanded time-scale. Accumulation and loss of clathrin begins in a fraction of a second, and the loss is delayed after the longer stimulus (arrows).

To investigate how the distribution of clathrin might be altered when a synapse is activated, we imaged the terminals of isolated bipolar cells using TIRFM (Figure 3A). The first aim was to characterize how clathrin might be recruited in relation to the active zone, so we used double transgenic zebrafish expressing clathrin LCa-EGFP and Ribeye-mCherry: Ribeye is the major protein component of the synaptic ribbon that holds vesicles close to the active zone (Schmitz, 2009). Supplementary Movie 1 shows LCa-EGFP in a synapse upon depolarization. At rest clathrin was present on many structures distributed close to the surface membrane, many of these were small and mobile, others larger and static. The predominant mobile membrane compartment in ribbon synapses are synaptic vesicles (Holt et al., 2004).

A depolarizing stimulus was found to cause two distinct types of change in the signal generated by LCa-EGFP: an accumulation of clathrin was observed in some areas of the terminal footprint, but a net loss was observed in others. An example of the accumulating response to a depolarizing stimulus lasting 3 s is shown in Figure 3B as the relative increase in fluorescence. A transient accumulation of clathrin was obvious around the two ribbons to the top of the footprint, which is shown in more detail in Figure 6B and in Supplementary Movies 2, 6. The average kinetics of clathrin accumulation are shown in Figure 3C, for two stimulus durations, 0.5 s (red dotted trace; *n* = 6 cells) and 3 s (red solid trace; *n* = 12 cells). Surprisingly upon stimulation clathrin starts to accumulate within one frame interval (100 ms) and was mostly complete within 1 s of the end of the stimulus (Figure 3D). The accumulation of clathrin near sites of synaptic vesicle fusion was therefore 2–3 orders of magnitude faster than the recruitment of clathrin to a coated pit during the classical mode of CME.

In other regions of the presynaptic membrane, depolarization triggered an immediate and transient *loss* of LCa-EGFP fluorescence, with average kinetics shown by the black traces in Figures 3C,D. These areas were not specifically associated with ribbons, but the time-scale on which clathrin left the evanescent field was broadly similar to the accumulating response. The loss of LCa-EGFP might be explained by a transient dissociation of clathrin from membrane compartments close to the cell surface, or by a transient net movement of these compartments away from the surface: these two possibilities could not be distinguished by TIRFM.

### Imaging synaptic vesicle fusion and retrieval by TIRF

**Figure 4:**
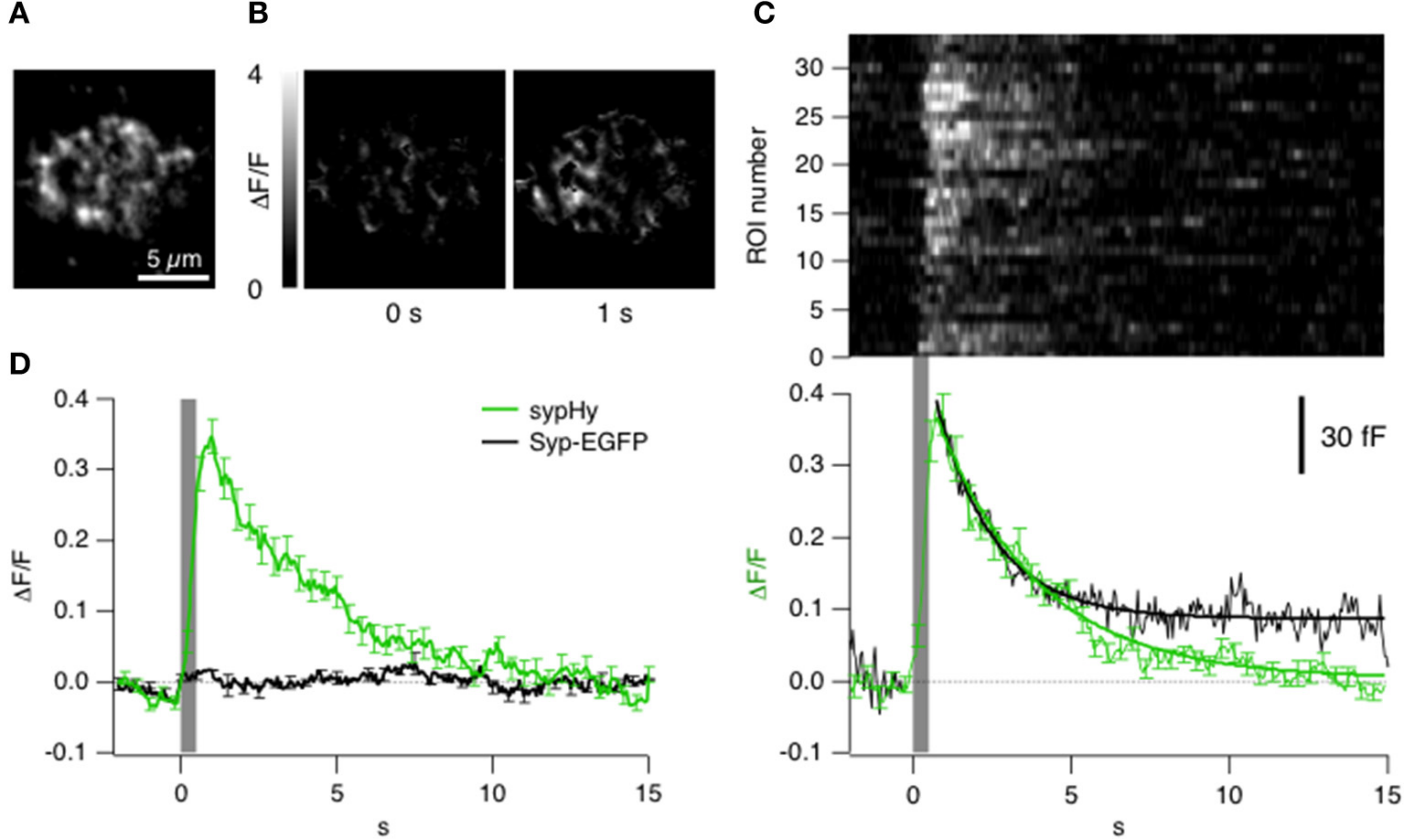
**Kinetics of vesicle fusion and retrieval observed with sypHy. (A)** Average fluorescence projection (before stimulation) of TIRF image of the synaptic terminal of a bipolar cell expressing sypHy. **(B)** Images showing the relative changes in fluorescence at different time points from the start of a stimulus lasting 0.5 s. These images have been backgroud subtracted and it was calculated the Δ*F*/*F* (variation of fluorescence to the F0 = basal fluorescence). **(C)** Top: Raster plot showing the kinetics of the sypHy signal in 33 ROIs from the footprint in **(B)**. Note the variability in time-course of recovery. Bottom: The average kinetics of the sypHy signal triggered by the same stimulus (green, 7 terminals). The black trace shows the time-course of the capacitance signal measured in zebrafish bipolar cell terminals in response to a 0.5 s depolarization (*n* = 3). **(D)** Comparison of the sypHy signal (green; 3 terminals) and synaptophysin-EGFP signal (black; 2 terminals) triggered by a 0.5 s stimulus. Traces in **(C,D)** were normalized to the maximum intensity of relative change in fluorescence. All of the sypHy signal can be attributed to vesicle fusion and subsequent events.

To understand the significance of the clathrin signals in relation to synaptic vesicle fusion and retrieval we used sypHy, a pHluorin-based reporter that signals the pH-changes experienced by the interior of a vesicle (Miesenböck et al., 1998). First we asked how far a sypHy signal observed by TIRFM might reflect these processes. Figures 4A,B show the sypHy response in a terminal stimulated for 0.5 s. “Hotspots” of fluorescence appeared within the evanescent field, reflecting regions where synaptic vesicles fused with the surface membrane (see Supplementary Movie 3). A kymograph displaying the relative change in fluorescence in 33 separate regions of interest (ROIs) in this footprint is shown in Figure 4C. The sypHy signal reached a maximum ~300 ms after the end of the stimulus and then immediately recovered with a time-constant of 3.2 ± 0.1 s. Over a population of 7 cells, the decline in the sypHy signal was best described as a double exponential function, with 30% of the fluorescence recovering with a time-constant of 2.5 ± 0.8 s, and the remainder with a time-constant of 13.0 ± 0.5 s (Figure 4D, green trace).

A change in pHluorin intensity observed by TIRFM might be caused by movement of the fluorophore in a direction normal to the membrane or out of the static ROI, as well as by changes in intravesicular pH. It was therefore necessary to test whether the sypHy signal might also reflect movements of internal membrane compartments carrying synaptophysin, which we did by imaging bipolar cells expressing synaptophysin-EGFP. The EGFP was on the cytoplasmic side of the vesicle so that the fluorophore was always subject to cytoplasmic pH. Depolarization did *not* generate a significant change in the total amount of synaptophysin under the membrane, indicating that there was little net change in the number of vesicles within the evanescent field (Figure 4D, black trace) and that the sypHy transients could be attributed to the processes of fusion and followed by endocytosis. As a further test of the idea that the decline in the sypHy fluorescence reflected endocytosis we made separate capacitance measurements in bipolar cells from zebrafish. The decline of sypHy and capacitance signals after a depolarization lasting 0.5 s occurred with a very similar time-course (Figure 4C, black trace). We were therefore able to use sypHy to monitor both exocytosis and endocytosis while observing the accumulation and loss of clathrin (below). Strictly, a decline in sypHy fluorescence reflects reacidification of vesicles and/or their movement away from the surface, but both these processes only occur *after* scission of the vesicle from the surface membrane.

### Clathrin dynamics in relation to synaptic vesicle fusion and retrieval

**Figure 5:**
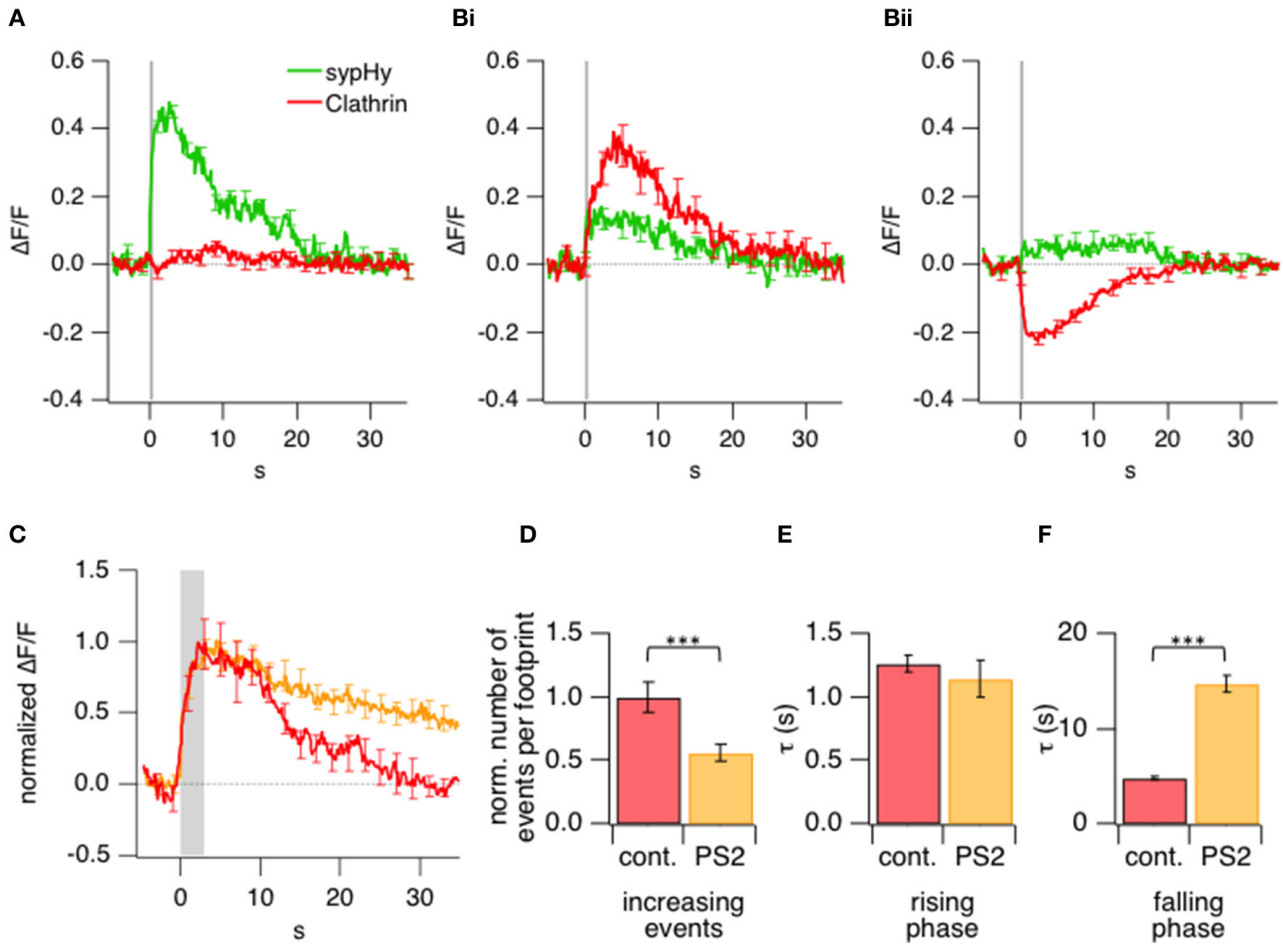
**Kinetics of exo- and endocytotic events in relation to clathrin accumulation and loss. (A)** Comparison of the average sypHy signal (green) and LCa-mCherry signal (red) measured simultaneously in ROIs selected by detecting a significant increase in sypHy fluorescence during a 0.5 s stimulus. 615 ROIs from 16 terminals. **(B)** Comparison of the average sypHy signal (green) and LCa-mCherry signal (red) measured simultaneously in ROIs selected according to a rise **(i)** or fall **(ii)** in the clathrin signal within the first 10 s (cf. Figure 3C). 438 ROIs from 16 terminals. **(C)** Average normalized timecourse of clathrin accumulation under control conditions (red) and in presence of the 30 μM clathrin inhibitor Pitstop2 (orange) triggered by stimuli lasting 3 s. **(D)** Number of events observed at the footprint before and after treatment with Pitstop2 upon 3 s stimulation, which led to a highly significant reduction in initially increasing events. Control: 203; Pitstop2: 109; 10 terminals **(E)** Kinetics of the rising phase of increasing events, which remained unchanged. **(F)** Kinetics of the falling (recovery) phase of increasing events which were significantly slowed down. (^***^P < 0.001).

Having established that sypHy could be used to monitor exocytosis and endocytosis by TIRFM, we went on to combine these measurements with simultaneous measurements of clathrin accumulation using LCa-mCherry. A key step in the analysis of these experiments was the method of defining the ROI over which fluorescence signals were measured on applying a stimulus. ROIs were defined as regions of the footprint in which the sypHy signal increased significantly (>4× SD of baseline fluorescence) in response to a 0.5 s stimulus. These sites of vesicle fusion did *not* accumulate LCa-mCherry, indicating that there was no net recruitment of clathrin at the immediate sites of vesicle fusion (Figure 5A).

An alternative way of analyzing the same experiments was to define ROIs according to the signal generated by LCa-mCherry at sites in which there was a significant increase or decrease (>4× SD of baseline fluorescence) in its fluorescence. These regions were identified by measuring the signal over a time-window of 10 s from the beginning of the stimulus so as to encompass the period of clathrin accumulation (Figure 5Bi) or loss (Figure 5Bii). Clathrin-accumulating ROIs also generated a sypHy signal, although weaker on average than those measured over ROIs defined from the sypHy signal alone. These results demonstrate that newly fused vesicular membrane tended to colocalize with sites of clathrin accumulation. In contrast, there was a much smaller sypHy signal in regions of the footprint where there was a significant loss of clathrin (Figure 5Bii), indicating that these areas were remote from sites of synaptic vesicle fusion and retrieval.

After the stimulus, LCa-mCherry was lost from sites of accumulation over a period of ~10–20 s (Figure 3C). Two observations indicated that this phase of the signal was related directly to endocytosis. First, the kinetics of LCa-mCherry loss mirrored the slow mode of endocytosis characterized in bipolar cells from goldfish using the capacitance technique (Von Gersdorff and Matthews, 1994; Neves and Lagnado, 1999; Heidelberger et al., 2002), which has been shown to be clathrin-dependent (Jockusch et al., 2005; Llobet et al., 2011). Second, the loss of clathrin was slowed by a factor of 3 by application of Pitstop2 (Figures 5C–F), which inhibits clathrin-dependent endocytosis by interfering with the binding of amphiphysin to the N-terminal domain (Von Kleist et al., 2011). Pitstop2 also caused a 1.8-fold reduction in the number of clathrin accumulation events per footprint (Figure 5D).

### Clathrin-dependent endocytosis occurs around the synaptic ribbon

**Figure 6:**
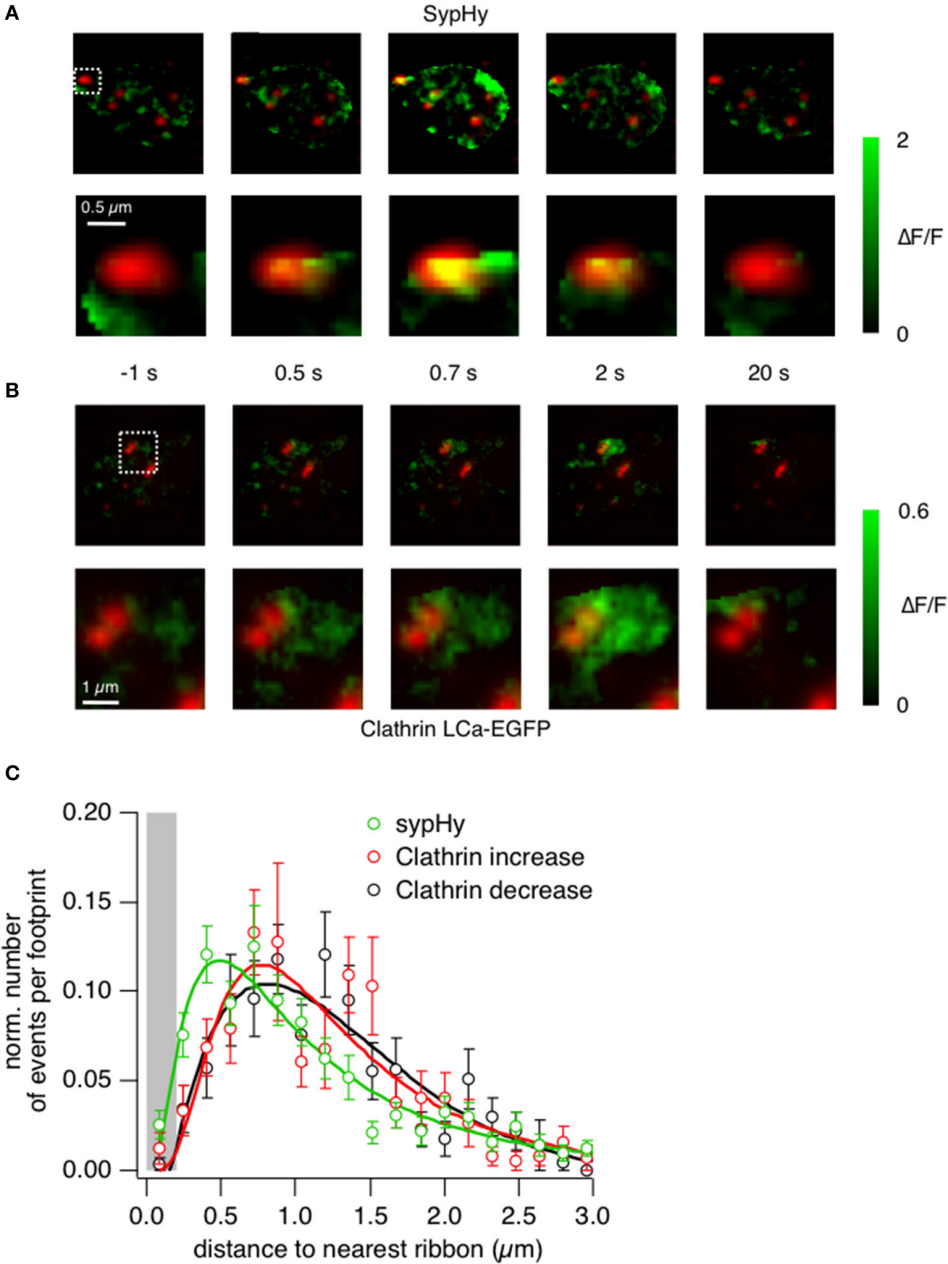
**Clathrin accumulates at the periphery of the active zone. (A)** TIRF imaging of the synaptic terminal of a bipolar cell expressing sypHy and ribeye-mCherry. Images show the relative change in fluorescence at times indicated relative to the start of a depolarizing stimulus lasting 0.5 s. The lower sequence is zoomed into the area marked by the square. Note that the signal generated in the first 0.5 s is closely localized to the active zone. **(B)** TIRF imaging of clathrin LCa-EGFP and ribeye-mCherry. The lower sequence is zoomed into the area marked by the square. Images obtained at the times corresponding to the experiment in **(A)**. Note that the signal generated in the first 0.5 s is around the active zone rather than immediately under it. **(C)** The position of vesicle fusion events (green) and regions of clathrin accumulation (red), relative to the center of the ribbon. The average radius of a ribbon was ~200 nm (as shown by the gray bar). Note that clathrin tends to accumulate and disappear further away than the initial sites of vesicle fusion.

Further evidence that shows an enrichment of clathrin at the active zone was provided by closer analysis of the sypHy and clathrin signals. The example images in Figure 6A show the location of sypHy signals with reference to ribbons marked by ribeye-mCherry, with the second row zoomed in on one particular active zone (Supplementary Movies 4, 5 and Supplementary Figures 2A–F). Vesicle fusion during the 0.5 s stimulus begins under the ribbon (frame at *t* = 0.5 s), after which some fused vesicular membrane also appears in regions around it. In contrast, an accumulation of clathrin-EGFP is *not* apparent under ribbons during a 0.5 s stimulus (Figure 6B), but within 2 s it can be clearly observed in surrounding regions (Supplementary Movie 6). Interestingly at higher magnification it was possible to observe quite static “patches” of clathrin surrounding the ribbons even before the stimulus onset (Figure 6B, box magnified). This might suggest that clathrin is immobilized and bound to structures surrounding the ribbons such as endosomes or cisternae or docked vesicles.

The locations of fusion events and clathrin accumulation relative to the active zone are shown in Figure 6C, collected from a total of 161 ribbons in 38 cells. The position of the active zone was defined as the center of mass of the nearest punctum of ribeye-mCherry. The location of sypHy events (also measured as the center of mass of the corresponding ROI) had a distribution that peaked ~400 nm from the center of the ribbon, while recruitment and loss of clathrin peaked at a distances of ~800 nm (Figure 6C). All three distributions had a long tail, consistent with the observation that synaptic vesicles can also fuse at sites remote from the ribbon, although at lower rates (Zenisek et al., 2000; Midorikawa et al., 2007). The displacement between the two distributions shown in Figure 6 is consistent with the idea that vesicular membrane travels roughly 400 nm away from the ribbon within about 1 s of fusion.

The location of accumulated membrane that we observed in live cells (Figure 6) resembles the changes in ultrastructure observed by electron microscopy by Holt et al. (2004), where large tubular invaginations of the surface membrane and cisternae/endosomes accumulate in regions around synaptic ribbons following stimulation (Paillart et al., 2003; Matthews and Sterling, 2008). We therefore suggest that these cisternae and/or endosomes are the primary structures associated with the transient accumulation of sypHy and clathrin in the region around the synaptic ribbon.

**Figure 7:**
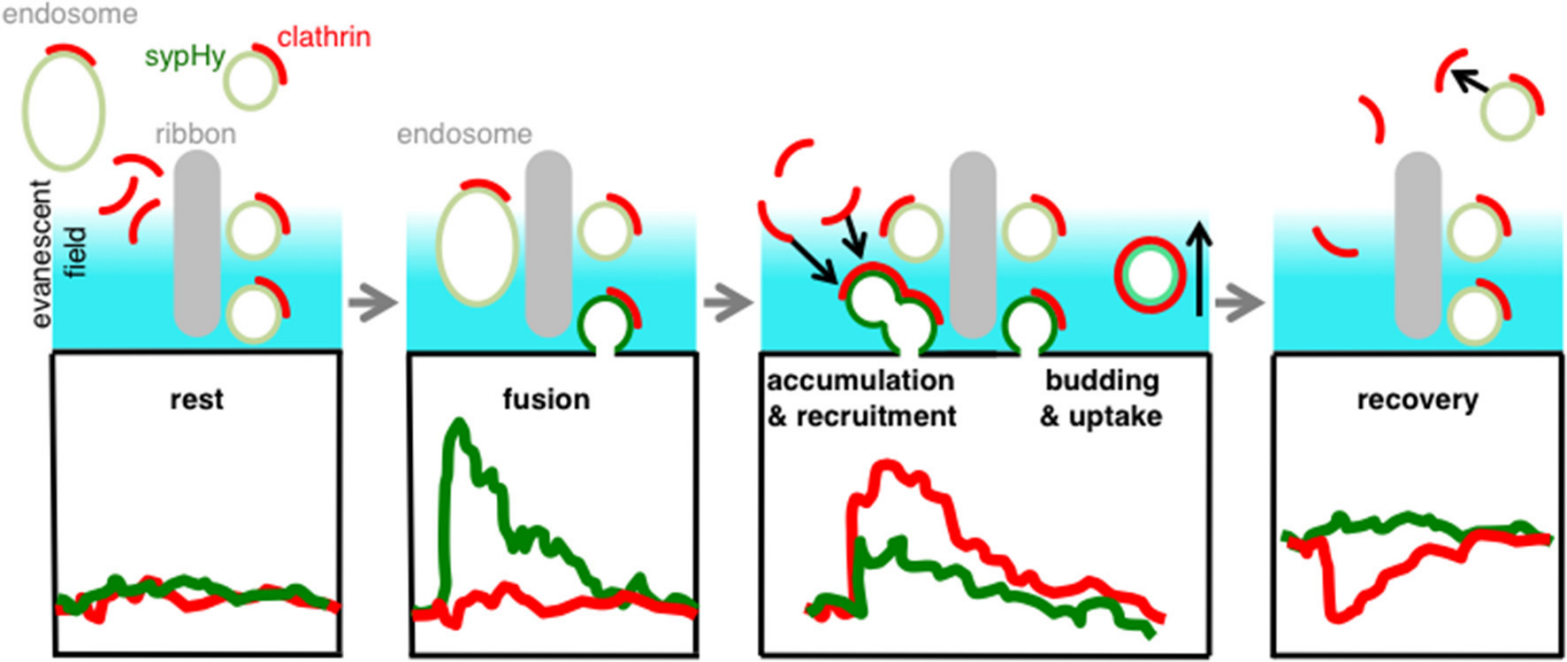
**Schematic model**. Clathrin is immobilized on docked-vesicles and endosomes/cisternae at the active zone. When a vesicle fuses we observe a clear SypHy signal (green trace) but no net signal of clathrin (red), because the net recruitment and accumulation occurs while the sypHy signal is already decaying (red trace). During the uptake of the vesicle we observe a decay of clathrin signal indicating that these areas were remote from sites of synaptic vesicle fusion and retrieval. With TIRF imaging we were able to discriminate at different hotspots all steps of vesicle recycling.

The picture that emerges from these results is one in which the accumulation and loss of clathrin at the surface membrane occurs with kinetics similar to the accumulation and retrieval of vesicular membrane. There was no measurable delay between the fusion of the synaptic vesicle and the appearance of clathrin around the active zone, consistent with the results in Figures 1, 3. These observations can be reconciled by the model shown in Figure 7. We suggest that docked synaptic vesicles and/or endosomes surrounding the ribbon carry clathrin, and that there is no further significant recruitment of clathrin directly at the active zone when exocytosis is triggered (cf. Figure 3A). However, the excess vesicular membrane then quickly spreads to the region neighboring the active zone, carrying both clathrin and unquenched sypHy (Figure 3B). Subsequently, vesicular membrane carrying clathrin and unquenched sypHy is retrieved by endocytosis.

## Discussion

It has been generally assumed that the clathrin-dependent retrieval of synaptic vesicles occurs through mechanisms broadly similar to classical CME of surface receptors, and that recruitment of clathrin occurs after fusion has occurred (Granseth et al., 2007; Mcmahon and Boucrot, 2011; Saheki and De Camilli, 2012). Here we have shown that clathrin is present at rest most probably bound or immobilized on docked vesicles or endosomes budding new vesicles. This idea was supported by the high degree of colocalization between clathrin and synaptic vesicles (Figure 1C), the presence of clathrin over large areas of the terminal footprint under TIRF (Figure 2A), the measurements of clathrin mobility using TIRF–FRAP, FCS and FCCS (Figures 2B–D) and the sub-second delay to clathrin accumulation and loss on triggering exocytosis (Figure 3C). These observations help us understand why clathrin-dependent retrieval of synaptic vesicles is so much faster than the formation of clathrin-coated pits during classical CME of surface receptors: synaptic vesicles are “primed” with clathrin so that membrane retrieval is not rate-limited by recruitment.

### How is clathrin involved in endocytosis at a synapse?

It has long been recognized that efficient mechanisms of membrane retrieval are required to maintain neurotransmission (Takei et al., 1996; Saheki and De Camilli, 2012). Here we have visualized the actual dynamics of synaptic vesicles in and around the active zone and find that the stimulation of fusion is followed within a fraction of a second by an accumulation of membrane in the surrounding region, and this membrane carries both synaptophysin and clathrin (Supplementary Movies 1–6). These dynamic observations can be compared to static images of endocytic proteins made at the ribbon synapse of photoreceptors with immunogold-EM by Wahl et al., who found that dynamin, syndapin, and amphiphysin are all enriched in the region around the active zone and the synaptic ribbon, and more importantly that clathrin could be found localized at the ribbons themselves (cf. Figure 12; Wahl et al., 2013). Similarly, ultrastructural observations at the conventional synapses of hippocampal neurons indicate that compensatory endocytosis occurs within ~100 ms at sites flanking the active zone rather than the active zone itself (Watanabe et al., 2013b). These studies, together with our own, demonstrate that the periactive zone is the major site of fast endocytosis at the synapse. The present study goes further by showing that clathrin is moving with a slow diffusion coefficient which is not compatible with the state of “free” molecules at ribbon synapses. Clathrin is carried along with vesicular membrane of endosomes toward the region around the active zone after synaptic vesicles fuse.

It seems very unlikely that a large proportion of this clathrin is organized into a “cage” because electron microscopy reveals relatively few coated vesicles after a ribbon synapse is stimulated (Matthews and Sterling, 2008; Llobet et al., 2011). The most obvious structure to appear around the ribbon are large uncoated invaginations, including tubules connected to the surface (Paillart et al., 2003; Holt et al., 2004; Matthews and Sterling, 2008). How, then, is clathrin involved in the retrieval of synaptic vesicles? Two kinetically-distinct modes of endocytosis have been recognized at the ribbon synapse of retinal bipolar cells: fast (~1–2 s) and slow (~10–15 s) (Neves and Lagnado, 1999). The slow mechanism is disrupted by injection of peptides that block interactions between clathrin and the adaptor complex AP2, indicating that this core aspect of classical CME is retained (Jockusch et al., 2005). The fast mechanism of endocytosis is insensitive to these manipulations, indicating that it is distinct from other clathrin-dependent pathways. We cannot, however, rule out the possibility that clathrin is also involved in fast endocytosis, because the delay between stimulation and the accumulation of clathrin around the active zone was just a fraction of a second (Figure 3D). For instance, it may be that this fast mechanism uses a less complex molecular machinery to form an invagination of the surface membrane and a neck at which scission occurs through the action of dynamin. A molecule likely to play a key role in this faster processes is endophilin, which both bends membrane through an N-BAR domain and recruits dynamin through an SH3 domain (Gallop et al., 2006), and has been shown to get involved into endocytosis already in the stage of exocytosis (Bai et al., 2010). Indeed, interfering with the function of endophilin selectively blocks the fast mode of endocytosis in bipolar cell terminals (Llobet et al., 2011). Images obtained by STED microscopy demonstrate that a number of proteins involved in endocytosis are associated with synaptic vesicles (Denker et al., 2011) and the idea is that the reserve pool of vesicles acts as a “buffer” system for proteins involved in endocytosis. So it may be that “priming” for fast endocytosis involves not only clathrin, but also other proteins with which it interacts. Recent findings indicate that at hippocampal synapses vesicles can be retrieved in 50–100 ms by a mechanism that is neither compatible with “kiss-and-run” nor with CME, which is called “ultrafast” endocytosis (Watanabe et al., 2013b). However, the role of clathrin in this process remains still controversial. It has been shown that clathrin is required to retrieve synaptic vesicles from the endosomes and not from the plasma membrane at central synapses (Kononenko et al., 2014; Watanabe et al., 2014). At the specialized synapses recent findings show clearly that CME is the dominant mechanism of retrieval at the mouse bipolar synapses. In contrast, at the photoreceptor terminals is predominant a clathrin-independent mechanism of retrieval, promoted by very long stimuli (3–15 h) and other molecules such as dynamin3, endophilin, and synaptojanin (Fuchs et al., 2014).

In summary this just shows how controversial CME is still discussed after nearly 40 years of clathrin's discovery (Pearse, 1976). The fast retrieval we are observing in this paper resembles ultrafast endocytosis described by Watanabe et al. (2014). However, in the Watanabe paper, brief light stimuli were given (<300 ms). Our stimulus paradigm sits right in the middle between the ultrafast and the “bulk” endocytosis. The possible scenario we are proposing in our model is: at rest clathrin is bound to docked vesicles or endosomes/cisternae surrounding the ribbon, when a stimulus occurs clathrin or membrane patches carrying clathrin are recruited in less than 100 ms fusing to the plasma membrane and accumulating for 3 s. This is compatible to the changes in ultrastructure observed by electron microscopy by Holt et al. (2004), where large tubular invaginations of the surface membrane and cisternae/endosomes accumulate in regions around synaptic ribbons following stimulation. In summary we observed that at bipolar synapses the fast retrieval is clathrin-dependent and this might be promoted by a “priming” step of the ribbon-docked synaptic vesicles. Further investigations are needed to understand more in details the biochemical nature of this “priming” mechanism. It is probable that the classical machinery involved in CME is modified at the ribbon synapses and does require a fast recycling of vesicles and clathrin molecules ready-to-use already on site.

### Conflict of interest statement

The authors declare that the research was conducted in the absence of any commercial or financial relationships that could be construed as a potential conflict of interest.

## Supplementary material

Supplementary Figure 1 **(A)** Normalized intensity vs. distance distribution through the equatorial plane of three representative bipolar cell terminals from two different transgenic zebrafish lines. The cell from the double transgenic synaptophysin-EGFP and clathrin-LCa-mCherry shows similar distribution pattern emphasizing the edges of the cell, whereas the cell from the dynamin2-EGFP transgenic zebrafish shows a uniform distribution. **(B)** Confocal images of the representative cells used in **(A)**. Intensities derived from areas underlying the dotted line. Scale bars: 2 μm.

Supplementary Figure 2 **Confocal imaging on live transgenic fish (6 dpf) obtained from a cross of the ribeye:sypHy with the ribeye:ribeye-mCherry line. (A)** SypHy expression is specifically located at the bipolar cell terminals in the inner plexiform layer (IPL). Weak fluorescence is also detected around bipolar cell soma in the inner nuclear layer. **(B)** Puncta of ribeye-mCherry marking ribbons in bipolar cell terminals in the IPL. Some puncta may represent clusters of 2 or more ribbons (arrowheads). **(C)** Merge of **A,B**. Scale bar 5 μm. **(D–F)** Higher magnification of the area shown by the white dashed box. Scale bar 3 μm.

Supplementary Figure 3 **Calibration of FCS measurements with fluorescent species of different molecular weights. (A)** To determine the aspect ratio of the point spread function of the confocal microscope for use in equation 2.2 (see Materials and Methods), we measured a standard sample (Rhodamine 110 in water) in the FCS mode of the confocal microscope. Three concentrations were tested. R110 has a diffusion coefficient of 400 μm^2^ s^−1^ in water. The FCS probed volume (~0.11 μm^3^) had a radius of ~0.21 μm. **(B)** FCCS was performed in a solution containing both purified EGFP and mCherry. The two proteins had similar mobility (EGFP τ_*d*_ = 0.121 ms, *D* = 91 μm^2^ s^−1^, mCherry τ_*d*_ = 0.128 ms, *D* = 86 μm^2^ s^−1^) but no cross correlation between the two fluorescent proteins could be observed under this condition (orange trace). **(C)** FCS using HEK cells transiently expressing cytosolic EGFP, mRFP, and GluR2-EGFP, and using Rhodamine110, purified EGFP and mCherry in PBS. The relative mobilities of GFP and mRFP were similar but both were slower than in water (cytoEGFP τ_*d*_ = 0.46 ms, *D* = 23.9 μm^2^ s^−1^, cytomRFP τ_*d*_ = 0.51 ms, *D* = 21.6 μm^2^ s^−1^). No significant cross-correlation was observed between the signals in the red and green channels (not shown). **(D)** FCCS with a mixture of cyto-mRFP and GluR2-EGFP in HEK cells, where the diffusion coefficient of GluR2-EGFP in cytoplasm is 0.13 μm^2^ s^−1^. Barely any cross-correlation was observed. **(E)** Dual color FRAP (sequential protocol) performed in bipolar synaptic terminals, revealed similar diffusion coefficient between clathrinLCa-mCherry *D* = 0.007μm^2^ s^−1^ and synaptophysin GFP *D* = 0.0038 μm^2^ s^−1^.

Supplementary Table 1 **Sequences of primers used for generating transgenic zebrafish lines**.

Supplementary Movie 1 **Dynamics of clathrin LCa-EGFP around active zone**. TIRF imaging of the footprint of an isolated bipolar cell from the retina of a double transgenic fish expressing ribeye-mCherry to mark ribbons and clathrin LCa-EGFP. The timing of the depolarizing stimulus is marked by a white square in the top left-hand corner. Original movie acquired at 20 Hz but here every two frames have been averaged. The movie runs at 2× real-time. Note that clathrin is distributed throughout the evanescent field and that some is associated with slowly moving structures.

Supplementary Movie 2 **Dynamics of clathrin LCa-EGFP around active zone**. The same footprint as in Supplementary Movie 1, but the signal in the green channel represents the relative change in the fluorescence, with black at Δ*F*/*F* = 0, and saturation at Δ*F*/*F* = 0.6 (corresponding to Figure 3B). Note the transient appearance of clathrin LCa-EGFP around several (but not all) ribbons.

Supplementary Movie 3 **Dynamics of exocytosis and endocytosis imaged using sypHy**. TIRF imaging of the footprint of an isolated bipolar cell from the retina of a transgenic fish expressing sypHy. The signal marks the relative change in the fluorescence, with black at Δ*F*/*F* = 0, and saturating white at Δ*F*/*F* = 4 (corresponding to Figure 4B). The timing of the depolarizing stimulus is marked by a white square in the top left-hand corner. Original movie acquired at 20 Hz but here every two frames have been averaged.

Supplementary Movie 4 **Dynamics of sypHy signals in relation to active zones**. TIRF imaging of the footprint of an isolated bipolar cell from the retina of a transgenic fish expressing sypHy and ribeye-mCherry. The signal marks the relative change in the fluorescence, with black at Δ*F*/*F* = 0, and saturating white at Δ*F*/*F* = 2 (corresponding to footprint in Figure 3A). The timing of the depolarizing stimulus is marked by a white square in the top left-hand corner. The movie runs at 2× real-time.

Supplementary Movie 5 **Dynamics of sypHy signals in relation to an active zone**. Close-up of the ribbon shown in the top left of Movie 4 and in the lower part of Figure 6A. The timing of the depolarizing stimulus is marked by a white square in the top left-hand corner. The movie runs at 2× real-time. Note that the stimulus immediately generates a sypHy signal under the ribbon.

Supplementary Movie 6 **Dynamics of clathrin signals in relation to an active zone**. Close-up of the pair of ribbons shown at the top of Movie 1 and in Figure 3B. The timing of the depolarizing stimulus is marked by a white square in the top left-hand corner. The movie runs at 2× real-time. Note that the stimulus generates a delayed accumulation of clathrin LCa that begins in an area to the periphery of the ribbon.
