# Supplementary figures and images for: Synaptic vesicles are “primed” for fast clathrin-mediated endocytosis at the ribbon synapse
Source: Front Mol Neurosci. 2014 Dec 1;7:91. doi: 10.3389/fnmol.2014.00091 (PMC4248811; doi:10.3389/fnmol.2014.00091)

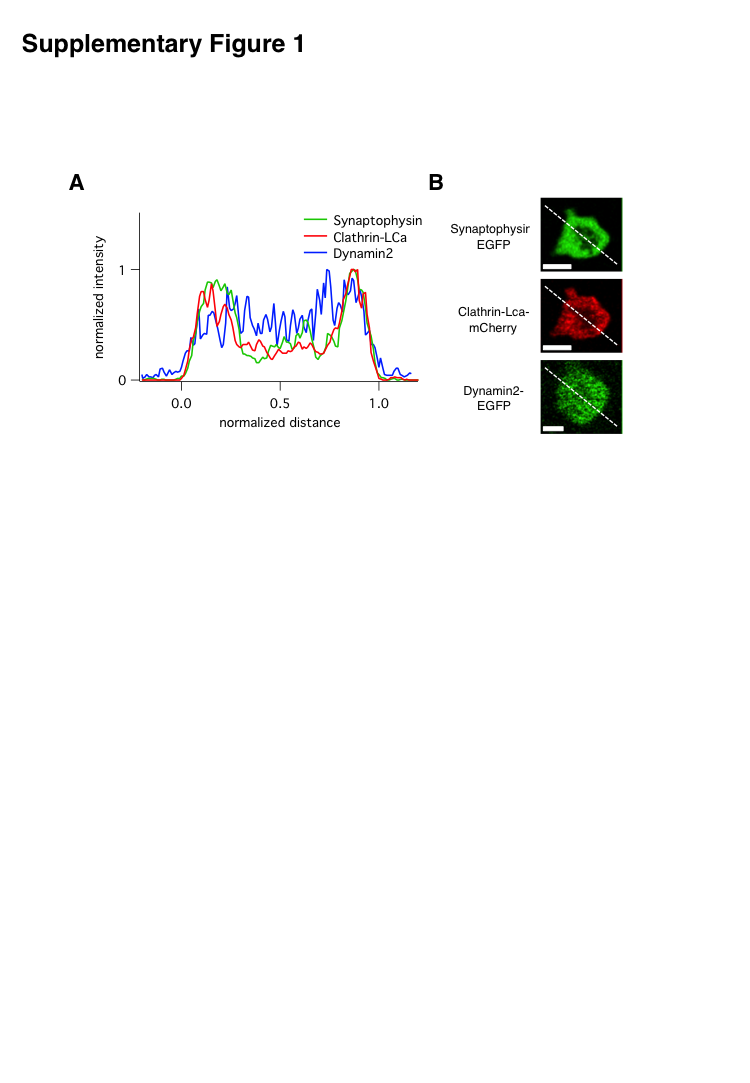

Supplement: Supplementary Figure 1 — (A) Normalized intensity vs. distance distribution through the equatorial plane of three representative bipolar cell terminals from two different transgenic zebrafish lines. The cell from the double transgenic synaptophysin-EGFP and clathrin-LCa-mCherry shows similar distribution pattern emphasizing the edges of the cell, whereas the cell from the dynamin2-EGFP transgenic zebrafish shows a uniform distribution. (B) Confocal images of the representative cells used in (A). Intensities derived from areas underlying the dotted line. Scale bars: 2 μm. [file Image1.TIF]

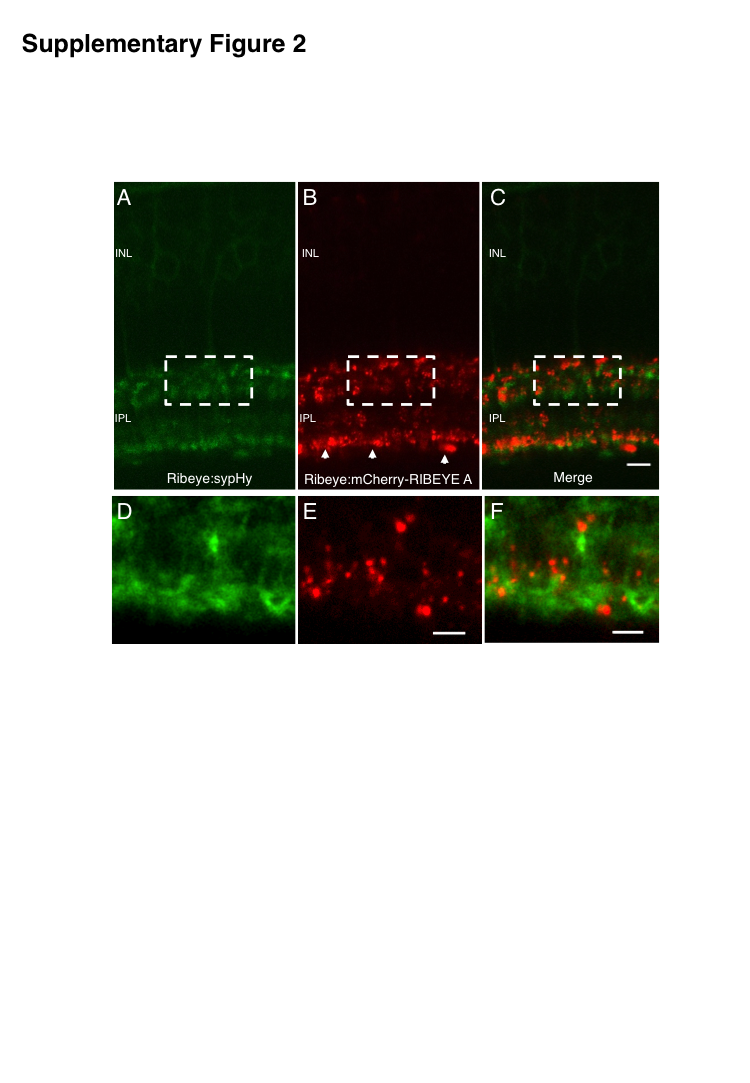

Supplement: Supplementary Figure 2 — Confocal imaging on live transgenic fish (6 dpf) obtained from a cross of the ribeye:sypHy with the ribeye:ribeye-mCherry line. (A) SypHy expression is specifically located at the bipolar cell terminals in the inner plexiform layer (IPL). Weak fluorescence is also detected around bipolar cell soma in the inner nuclear layer. (B) Puncta of ribeye-mCherry marking ribbons in bipolar cell terminals in the IPL. Some puncta may represent clusters of 2 or more ribbons (arrowheads). (C) Merge of A,B. Scale bar 5 μm. (D–F) Higher magnification of the area shown by the white dashed box. Scale bar 3 μm. [file Image2.TIF]

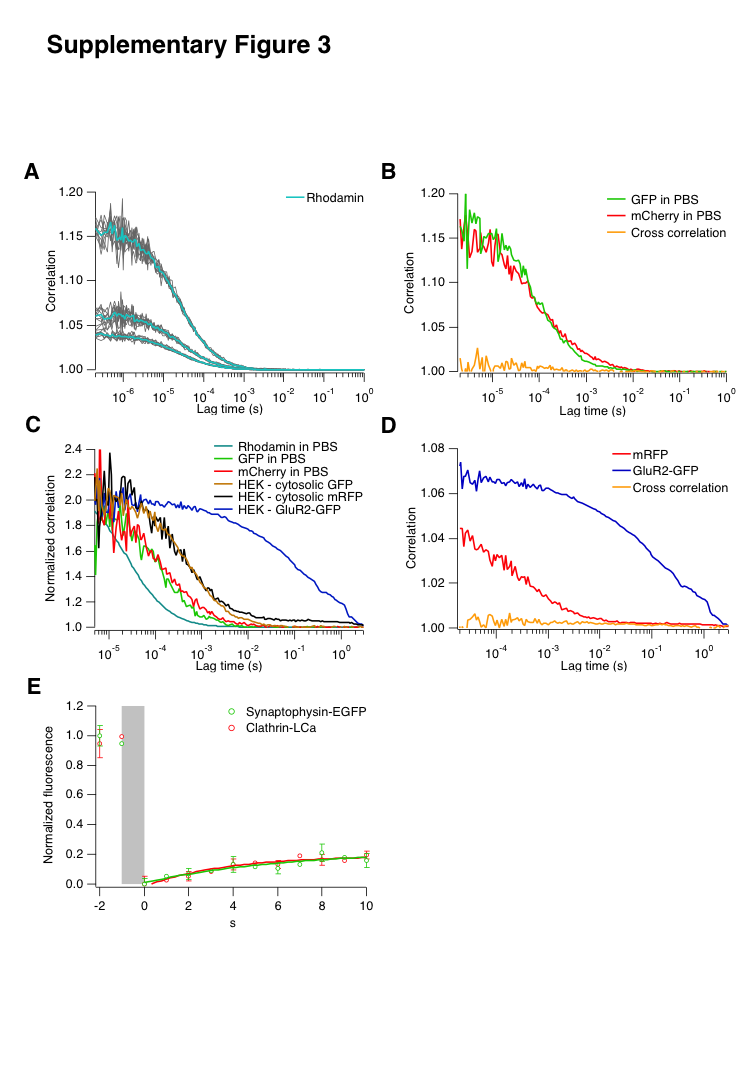

Supplement: Supplementary Figure 3 — Calibration of FCS measurements with fluorescent species of different molecular weights. (A) To determine the aspect ratio of the point spread function of the confocal microscope for use in equation 2.2 (see Materials and Methods), we measured a standard sample (Rhodamine 110 in water) in the FCS mode of the confocal microscope. Three concentrations were tested. R110 has a diffusion coefficient of 400 μm2 s−1 in water. The FCS probed volume (~0.11 μm3) had a radius of ~0.21 μm. (B) FCCS was performed in a solution containing both purified EGFP and mCherry. The two proteins had similar mobility (EGFP τd = 0.121 ms, D = 91 μm2 s−1, mCherry τd = 0.128 ms, D = 86 μm2 s−1) but no cross correlation between the two fluorescent proteins could be observed under this condition (orange trace). (C) FCS using HEK cells transiently expressing cytosolic EGFP, mRFP, and GluR2-EGFP, and using Rhodamine110, purified EGFP and mCherry in PBS. The relative mobilities of GFP and mRFP were similar but both were slower than in water (cytoEGFP τd = 0.46 ms, D = 23.9 μm2 s−1, cytomRFP τd = 0.51 ms, D = 21.6 μm2 s−1). No significant cross-correlation was observed between the signals in the red and green channels (not shown). (D) FCCS with a mixture of cyto-mRFP and GluR2-EGFP in HEK cells, where the diffusion coefficient of GluR2-EGFP in cytoplasm is 0.13 μm2 s−1. Barely any cross-correlation was observed. (E) Dual color FRAP (sequential protocol) performed in bipolar synaptic terminals, revealed similar diffusion coefficient between clathrinLCa-mCherry D = 0.007μm2 s−1 and synaptophysin GFP D = 0.0038 μm2 s−1. [file Image3.TIF]
