# Supplementary material for: Synaptic vesicles are “primed” for fast clathrin-mediated endocytosis at the ribbon synapse
Source: Front Mol Neurosci. 2014 Dec 1;7:91. doi: 10.3389/fnmol.2014.00091 (PMC4248811; doi:10.3389/fnmol.2014.00091)
Supplement: Supplementary Table 1 — Sequences of primers used for generating transgenic zebrafish lines. [file Table1.DOCX]

**Supplementary Table 1:**

| **Primer name:** | **Primer sequence 5’ to 3’:** |
| --- | --- |
| Zf clathrin forward | cac act cga gcc acc atg gac gat ttc gat atg ctc agt gc |
| Zf clathrin reverse | gaa gga tcc ccg cgg acg agc gga gcc tgt tta ag |
| Zf synaptophysin forward | cgc tag ctc gag cca cca tgg atg ttg cca acc agt tgg tcg cc |
| Zf synaptophysin reverse | gat gga tcc tcg ttg gag aag gat gtg ggc tc |
| Zf ribeye a forward | cat cat ctc gag cta tgt tga tct cca gta agc agt tgc cg |
| Zf ribeye a reverse | cat cat aga att cga tta ggt ata cat ttt gtc ttg cag gcc gcc |
| Zf dynamin2 forward | cat cat cac gtg cgc cac cat ggg caa ccg ggg gat gga gg |
| Zf dynamin2 reverse | cat cat acc ggt ggc ctg gtt tca ggt atc cag ctg tcg |
